# Attributable risk of suicide for populations in Australia

**DOI:** 10.3389/fpsyt.2023.1285542

**Published:** 2024-01-08

**Authors:** Piumee Bandara, Andrew Page, Lennart Reifels, Karolina Krysinska, Karl Andriessen, Marisa Schlichthorst, Anna Flego, Long Khanh-Dao Le, Cathrine Mihalopoulos, Jane Pirkis

**Affiliations:** ^1^Translational Health Research Institute, Western Sydney University, Campbelltown, NSW, Australia; ^2^Centre for Mental Health, Melbourne School of Population and Global Health, The University of Melbourne, Parkville, VIC, Australia; ^3^Health Economics Group, School of Public Health and Preventive Medicine, Faculty of Medicine, Nursing and Health Sciences, Monash University, Melbourne, VIC, Australia; ^4^Deakin Health Economics, Institute for Health Transformation, School of Health and Social Development, Deakin University, Geelong, VIC, Australia

**Keywords:** population attributable fraction, population attributable risk, suicide, suicide prevention, Australia

## Abstract

**Objective:**

Each year approximately 3,000 Australians die by suicide. We estimated the population attributable risk for identified target populations to provide evidence on how much of the overall burden of suicide in the Australian population is experienced by each of them.

**Methods:**

We identified 17 demographic and clinical target populations at risk of suicide and calculated the population attributable fraction (PAF) using a single or pooled suicide risk and the proportional representation of each target population within Australia.

**Results:**

Large PAF estimates were found for men (52%, 95% confidence interval (CI) 51%–53%), people bereaved by suicide (35%, 95% CI 14%–64%), people with a mental health or behavioural condition (33%, 95%CI 17%–48%), people with a chronic physical condition (27%, 95%CI 18%–35%), adults aged 25–64 years (13%, 95%CI 12%–14%), LGB populations (9%, 95%CI 6%–13%), offenders (9%, 95%CI 8%–10%), and people employed in blue collar occupations (8%, 95%CI 4%–12%).

**Limitations:**

The PAF is limited by assumptions, namely, that risk factors are independent, and that the relationship between risk factors and outcomes are unidirectional and constant through time.

**Conclusions and implications for public health:**

Considerable reductions in the overall suicide rate in Australia may occur if risk factors are addressed in identified populations with large PAF estimates. These estimates should be considered as an adjunct to other important inputs into suicide prevention policy priorities.

## Introduction

Suicide is the leading cause of death among Australians aged 15–44 years. In 2021, over 3,000 people died by suicide in Australia (1). Despite consistent efforts to reduce suicide in Australia, the suicide rate has remained relatively stable over the last two decades (2).

Suicide prevention remains a national health priority and in recent years there has been substantial attention to and investment in suicide prevention. The Prime Minister’s National Suicide Prevention Adviser has spearheaded a new focus on a whole-of-government approach for suicide prevention to comprehensively address the social, economic, health, cultural, and environmental factors contributing to suicide risk in the population (3). This approach recognises that specific populations are disproportionately impacted by suicidal behaviour and therefore warrant particular policy and research attention.

One way of examining the extent to which certain populations are at heightened risk is to calculate the population attributable fraction (PAF). The PAF estimates the proportion of cases of a particular health issue (e.g., suicide) that would be prevented if the effects of certain risk factors were comprehensively addressed (4). The PAF takes into consideration both the relative risk and the underlying prevalence of the risk factor in the population. This is important since risk factors with high relative risk but low prevalence, may have similar attributable risk to risk factors with lower relative risk but high prevalence (5). However, there are key assumptions of the PAF, namely, that the risk factors are independent, and that the relationship between risk factors and outcomes are unidirectional and constant through time (6, 7). In practical terms, these theoretical assumptions are not always met however, despite this, PAF estimates are a parsimonious and relatively objective way of quantifying risk that can complement other considerations to guide policy in particular areas.

Given the PAF assumes elimination of the risk factor will result in reductions in the outcome, studies have predominantly focused on modifiable clinical and social risk factors. A review of PAF used in suicidology showed psychiatric disorders are commonly identified as a large contributor to the risk of suicide and self-harm in high-income countries (4). A recent 2019 systematic review on the association between psychiatric disorders and suicide showed a PAF up to 21% for psychiatric disorders (8). Previous self-harm has also been identified as a significant contributor to self-harm in older adults in China (35%) (9) and in repeat self-harm (41%) (10), although the PAF of previous self-harm has not been examined, to our knowledge, for suicide mortality. In addition, unemployment and low socioeconomic position have been investigated as a potential contributor to suicide risk, with estimates ranging between 3% to 13% (4). The contribution appears to be larger among males, with a systematic review indicating an attributable suicide risk of 33% for manual labour/‘blue collar’ occupational status (5). Although risk factor elimination is not viable when considering sociodemographic categories such as occupational status, it is helpful to identify which population groups have a high attributable risk so that suicide prevention strategies may be targeted to address the risk factors specific to these groups. To our knowledge, no studies have systematically utilised the PAF to examine the attributable risk of a range of demographic sub-populations (e.g., men, young people, rural populations) to overall suicide risk in Australia. Accordingly, we estimate the attributable risk (the PAF) of suicide mortality for 17 identified target populations in Australia to serve as an adjunct to other inputs into suicide prevention policy decision-making.

## Methods

### Target populations

There are various taxonomies for considering groups that warrant attention in suicide prevention. For practical reasons, we used a taxonomy from one of our previous projects that had been developed for a different purpose (i.e., to identify Australian research priorities for suicide prevention) (11, 12). This was adapted slightly to include some additional target populations that had both well-documented high suicide risk (13–16) and relatively high proportion in the Australian population (17). In total, we identified 17 target populations: (1) men; (2) young people (aged 24 years or less); (3) adults (aged 25–64 years); (4) older adults (aged 65 years or more); (5) people born overseas; (6) Aboriginal and Torres Strait Islander people; (7) people residing in rural or remote Australia [i.e., living outside of a major city according to Australian Statistical Geography Standard (18)]; (8) people with a mental health or behavioural condition; (9) people with a chronic physical health condition; (10) people who have previously attempted suicide; (11) current or ex-serving military personnel; (12) offender populations; (13) lesbian, gay, or bisexual (LGB) populations (we were unable to include transgender people due to insufficient population-based data); (14) people bereaved by suicide; (15) people from lower socioeconomic backgrounds; (16) people employed in manual trade or “blue-collar” occupations; and (17) unemployed people.

### Suicide risk estimates for target populations

To calculate the PAF for a given target population, a relative risk estimate is needed. To identify the best available evidence, we conducted a rapid review of the literature for studies on [or allowing for the extraction of) suicide mortality risk (e.g., rate ratio, risk ratio, odds ratio) for each target population (see Table 1 for sources of suicide mortality risk estimates]. Initially, we searched the bibliographic database Medline for meta-analyses published between January 1 1990 to March 31 2023, using medical subject headings (MeSH) and text terms related to suicide and identified target populations. The search was augmented by secondary searches of identified articles and Google Scholar. If no meta-analyses were available relating to a given target population, estimates were sought from Australian population-based studies, and if no Australian-based studies were available, estimates were sought from international population-based studies. We excluded studies if they did not consider any of the target populations of interest; did not measure suicide mortality as the outcome; or did not report any empirical measure of association. The literature search did not identify reliable meta-analyses or population-based studies specifically relating to men, young people, adults, older adults, and Aboriginal and Torres Strait Islander people. For these populations, we calculated suicide mortality risk estimates using the Australian Bureau of Statistics (ABS) Causes of Death data (35) and corresponding 2016 Census population denominator data to estimate the incidence rate ratios.

**Table 1:** Target populations for suicide prevention in Australia: relative risk (RR), prevalence in the population, and population attributable fraction.

Target population	RR (95% CI)*	Prevalence (%)	PAF % (95% CI)	RR sources	Prevalence estimate sources
Males (vs. females)	3.21 (3.16–3.28)	49.3 (49.3–49.4)	52.2 (51.5–52.8)	ABS causes of death (2017–2021)	ABS 2021 census
Young people: 15–24 years (vs. adults & older people)	0.88 (0.86–0.90)	30.2 (30.2–30.2)	−3.8 (−4.4--3.1)	ABS causes of death (2017–2021)	ABS 2021 census
Adults: 25–64 years (vs. young people & older people)	1.29 (1.26–1.30)	52.6 (52.6–52.6)	13.2 (12.4–14.0)	ABS causes of death (2017–2021)	ABS 2021 census
Older people > =65 years (vs. adults & young people)	0.76 (0.75–0.78)	17.2 (17.2–17.2)	−4.3 (−4.6--4.0)	ABS causes of death (2017–2021)	ABS 2021 census
Overseas-born	0.87 (0.72–1.04)	29.5 (29.5–29.5)	−4.2 (−9.0–1.2)	Pooled estimate of different overseas-born groups based on: Hjern and Allebeck (19), cohort study [Sweden]; Morrell et al. (20), cross-sectional study [Australia]; and Ide et al. (21), cross-sectional study [Australia].	ABS 2021 census
Aboriginal and Torres Strait Islander	1.96 (1.47–2.6)	3.2 (3.2–3.2)	2.9 (1.4–4.7)	ABS Causes of Death (2017)	ABS 2021 census
Gay, lesbian, bisexual	4.52 (3.27–6.26)	3.0 (3.0–3.0)	9.3 (6.1–13.3)	Pooled estimate based on: Qin et al. (22), case–control study [Denmark] Mathy et al. (23), cross-sectional study [Denmark]	ABS (24) general social survey
Low socioeconomic position (vs. high)	1.27 (1.00–1.61)	27.4 (27.3–27.4)	6.6 (−0.2–13.9)	Orri et al. (25), meta-analysis	ABS household wealth 2017–18; ERP Jun 2018 denominator [% lowest two quintiles of both equivalised disposable household income]
Employed in manual trade/blue-collar occupation (vs. general employed)	1.84 (1.46–2.33)	10.2 (10.2–10.2)	7.8 (4.4–11.8)	Allison Milner et al. (16), meta-analysis	ABS 2021 Census [% Labourers as proportion of total employed population]
Unemployed (vs. employed)	1.15 (1.00–1.13)	2.4 (2.4–2.4)	0.4 (0.0–0.7)	Allison Milner et al. (15), meta-analysis	ABS 6202.0 – Labour Force, Australia, Jan 2020
Rural or remote residence (vs. metropolitan)	1.07 (1.01–1.13)	28.6 (28.6–28.6)	2.0 (0.4–3.6)	Qi et al. (26), cross-sectional study [Australia]	ABS Regional Population Growth 2018 [% ERP Outside of Major City (ASGS)]
Bereaved by suicide (vs. no)	3.23 (2.32–4.51)	29.4 (20.7–39.0)	35.2 (14.0–63.9)	Hill et al. (36), meta-analysis	Andriessen et al. (27) Meta-analysis of population-based surveys
Mental or behavioural condition (vs. no)	2.49 (1.66–3.76)	33.8 (33.8–33.8)	32.6 (17.0–47.6)	Pooled estimate based on: Franklin et al. (13), meta-analysis Ribeiro et al. (28), meta-analysis Darvishi et al. (29), meta-analysis Gili et al. (30), meta-analysis	ABS 2020–21 National Study of Mental Health and Wellbeing
Physical chronic condition (vs. no)	1.78 (1.49–2.12)	47.3 (46.6–48.0)	26.8 (18.3–34.8)	Franklin et al. (13), meta-analysis	ABS 2017–18 National Health Survey
Previous suicide attempt (vs. no)	2.03 (1.61–2.57)	4.8 (4.1–5.5)	4.4 (2.2–8.7)	Ribeiro et al. (31), meta-analysis	ABS 2020–21 National Study of Mental Health and Wellbeing [lifetime prevalence]
Military personnel	1.79 (0.55–5.77)	2.4 (2.4–2.4)	1.0 (−1.5–9.6)	Pooled estimate based on: Bergman et al. (32), retrospective cohort study [Scotland] Milner et al. (33), case-series compared to population rate [Australia]	ABS 2021 Census [% defence personnel of working age population]
Offenders	6.76 (6.08–7.52)	1.8 (1.8–1.8)	9.3 (8.3–10.4)	Jones and Maynard (34), meta-analysis	ABS Recorded Crime – Offenders 2020–21

*CI=Confidence interval. Unless specified, reference population for comparison is the general population.

### Population proportion estimates for target populations

We ascertained estimates of the proportional representation of most of the target populations within the Australian population from the 2016 ABS Census data. We used specific sources (predominantly national surveys and registries) to determine the proportional representation of the following target groups: people with a chronic physical health condition and people currently managing via treatment or medication a mental health or behavioural condition (37), offender populations (38), LGB populations (24), current and ex-serving military personnel (39), people with a previous suicide attempt (37), and people bereaved by suicide (27) (Table 1). It is important to note that comparisons between PAF estimates can only be made for target groups from the same source population (i.e., with the same population denominator in the PAF calculation).

### Statistical analysis

Where no meta-analysis was identified for a given target population, we pooled the relative risk of suicide from population-based studies using a series of random effects meta-analyses. No meta-analysis was conducted for target populations where only a single effect size was identified. We then calculated PAF estimates for each target population using the single or pooled relative risk estimate and the estimated population proportion for each target population. We conducted meta-analyses in Stata (version 15.1), using the “metan” function to pool risk estimates. Monte-Carlo simulation models using Ersatz Software 1.35 (40) were used to estimate the 95% confidence intervals for PAF estimates, to account for the uncertainty around relative risk and population proportion estimates. These simulations allowed for multiple re-calculation of PAF estimates taken from randomly drawn values from the distributions defined for relative risk and proportion estimates. We used a beta probability distribution for population proportion estimates using the ErBeta function, and a normal distribution (for the natural logarithm of the relative risk) was used for relative risk estimates using the ErRelativeRisk function, to estimate 95% confidence intervals for PAF estimates after 10,000 iterations to ensure convergence of model outcomes.

## Results

Large PAF estimates for suicide in Australia were associated with men (52.2%, 95% CI 51.5%–52.8%), people bereaved by suicide (35.2%, 95%CI 14.0%–63.9%), people with a mental health or behavioural condition (32.6%, 95%CI 17.0%–47.6%), people with a chronic physical condition (26.8%, 95%CI 18.3%–34.8%), adults aged 25–64 years (13.2%, 95%CI 12.4%–14.0%), LGB populations (9.3%, 95%CI 6.1%–13.3%), offenders (9.3%, 95%CI 8.3%–10.4%), and people employed in blue collar occupations (7.8%, 95%CI 4.4%–11.8%) (Table 1). PAF estimates of 4.4% were estimated for people with a previous suicide attempt (95%CI 2.2%–8.7%) and 2.9% for Aboriginal and Torres Strait Islander populations (95%CI, 1.4%–4.7%) (Table 1).

## Discussion

The PAF is an objective and parsimonious metric to help guide policy decision-making, alongside other important inputs and considerations, such as stakeholder consultations, dynamic simulation modelling, equity criteria, and cost-effectiveness (41). The PAF estimates from the current study indicated sizable reductions in the overall Australian suicide rate if suicide risk is comprehensively addressed among men, people bereaved by suicide, people with a mental health or behavioural condition, people with a chronic physical condition, and the adult population. PAF estimates also indicated considerable reductions in suicide could be achieved if effective approaches were delivered for LGB populations, offenders, and people employed in blue collar occupations. The high PAF estimates of these populations indicate the need for the development and evaluation of targeted interventions.

Given the PAF is often applied to modifiable risk factors, evidence for most of the identified (largely static) populations is scarce. However, results for people with a mental or behavioural condition were consistent with a recent systematic review of psychiatric disorder and suicide mortality which estimated a PAF of 21% (8). PAF estimates for suicide associated with blue-collar occupation have been previously shown to be higher in males (33%) than females (7%) (5). Due to insufficient statistical power, it was not possible to conduct sex-stratified analyses, however, the lower overall PAF estimate for suicide associated with blue-collar occupations in the current study (8%) may be explained by the low proportional representation of blue-collar occupations in the Australian working population. In addition, the low overall PAF estimate for suicide associated with unemployment was consistent with previous studies which range between 2% for females to 4% for males (5). Further research is needed among other sub-populations to confirm the findings. It is also important to note that these groups are not necessarily mutually exclusive and are likely to also comprise those with mental or behavioural conditions and diverse socio-demographic groups (for example in terms of migrant status and other markers of socio-economic status).

Evidence is still emerging for interventions for the whole population, and very few studies have been conducted to determine whether certain interventions are differentially effective for particular groups (42, 43). In many other areas of health (e.g., heart disease), PAF estimates assume that a particular risk factor (e.g., smoking) is eliminated from the population. In the current study, where the risk factor is a specific target population, this assumption is clearly not appropriate; but we do want to address and mitigate the factors that place these populations at greater risk. If we can do this, considerable reductions in suicide may be made. As an example, the high PAF of 52% highlights the need to explore factors that place men at greater risk. A 2019 scoping review of suicide prevention strategies for men highlighted the importance of reframing help-seeking as masculine, offering support in informal settings by trusted individuals, and providing emotional regulation techniques (44).

Although PAFs are one input into policy decision-making, they represent a metric that should serve as an adjunct to other important considerations, including health inequities. Aboriginal and Torres Strait Islander people, despite representing a small proportion of the total Australian population (3%) and thus a having relatively small PAF, are twice as likely to die by suicide compared to the general population (45). There is a clear need to prioritise and tailor culturally appropriate suicide prevention research and resources for this population (46). Similarly, although young people have a relatively low PAF, they are disproportionately represented in hospital admissions for self-harm (47), indicating the importance of early intervention.

### Limitations

There are important methodological limitations that should be considered when interpreting the results. First, although the search strategy for relative risk estimates identified key meta-analyses and population-based studies, we did not conduct systematic reviews of individual studies relevant to each target population. It is possible that some key studies may have been missed which could have potentially biased the relative risk estimate used in calculating a given PAF. In addition, no formal quality assessment of the studies was conducted, therefore, sources of bias and confounding may affect the risk estimates used. Furthermore, the underlying population proportion estimate used to calculate the PAF is likely under-enumerated for some target populations (e.g., LGB groups and people with a previous suicide attempt), due to stigma and poor surveillance (48, 49). Additionally, although comparisons can be theoretically made between PAF estimates for target groups from the same source population (i.e., same population denominator in the PAF calculation), caution should be exercised. PAF estimates assume that relative risk and prevalence estimates are not affected by sources of bias or confounding, that exposure variables are independent, and relationships between exposures and outcomes are unidirectional, linear, and constant through time (6, 7, 50). These assumptions are difficult to uphold given the multifactorial nature of suicide risk, the likelihood that risk distributions will change over time, and the intersection and overlap of multiple exposures and identities across the target populations. Despite these limitations, PAF estimates can serve as an adjunct to other priority-setting exercises and an initial starting point prior to more sophisticated considerations.

### Conclusions and implications for public health

PAF estimates are one means of informing suicide prevention efforts and can augment other important inputs that are used in policy decision-making. Findings from the current study suggest that addressing determinants of suicide among men, people bereaved by suicide, people with a mental health or behavioural condition, people with a chronic physical condition, the adult population, LGB population, offenders, and people employed in a manual trade may contribute substantially to reductions in the overall suicide rate in Australia.

## Data availability statement

The original contributions presented in the study are included in the article/supplementary material, further inquiries can be directed to the corresponding author.

## Author contributions

PB: Conceptualization, Data curation, Formal analysis, Methodology, Writing – original draft, Writing – review & editing. AP: Conceptualization, Data curation, Formal analysis, Methodology, Writing – review & editing. LR: Conceptualization, Funding acquisition, Methodology, Supervision, Writing – review & editing. KK: Writing – review & editing. KA: Writing – review & editing. MS: Writing – review & editing. AF: Writing – review & editing. LL: Writing – review & editing. CM: Writing – review & editing. JP: Conceptualization, Funding acquisition, Methodology, Supervision, Writing – review & editing.
